# Discovery and validation of extracellular vesicle‐associated miRNAs as noninvasive detection biomarkers for early‐stage non‐small‐cell lung cancer

**DOI:** 10.1002/1878-0261.12889

**Published:** 2021-01-06

**Authors:** Yujie Zhong, Xiaoyu Ding, Yuying Bian, Jing Wang, Wanqing Zhou, Xiangdong Wang, Pumin Li, Yi Shen, Jun‐Jun Wang, Jing Li, Chunni Zhang, Cheng Wang

**Affiliations:** ^1^ Department of Clinical Laboratory Jinling Hospital State Key Laboratory of Analytical Chemistry for Life Science Nanjing University School of Medicine Nanjing University China; ^2^ State Key Laboratory of Pharmaceutical Biotechnology Jiangsu Engineering Research Center for MicroRNA Biology and Biotechnology NJU Advanced Institute for Life Sciences (NAILS) Nanjing University School of Life Sciences Nanjing University China; ^3^ Department of Laboratory Medicine Nanjing Drum Tower Hospital the Affiliated Hospital of Nanjing University Medical School China; ^4^ Department of Laboratory Medicine, the Affiliated Chest Hospital of Nanjing Medical School China; ^5^ Department of Thoracic Surgery Jinling Hospital Nanjing University School of Medicine Nanjing University China

**Keywords:** biomarker; early diagnosis, circulating miRNAs, extracellular vesicles, non‐small‐cell lung cancer

## Abstract

miRNAs in circulating extracellular vesicles (EVs) are promising biomarkers for cancer. However, their diagnostic ability for early‐stage non‐small‐cell lung cancer (NSCLC) is not well known. In this study, the circulating EV miRNAs profiling was initially performed in 36 untreated NSCLC patients and 36 healthy controls by TaqMan Low Density Array (TLDA). Subsequently, we performed quantitative reverse‐transcription PCR assay (RT‐qPCR) validation in several independent cohorts that included 159 NSCLC patients, 120 age/sex‐matched healthy controls and 31 benign nodule patients enrolled from three different clinical centres. In addition, 38 cases of NSCLC were analysed before and after surgery. We demonstrated that miR‐520c‐3p and miR‐1274b were significantly and steadily increased in NSCLC patients in comparison with healthy controls and benign nodule patients (*P* < 0.001) and decreased markedly after tumour resection (*P* < 0.001). The areas under the curve (AUCs) of the ROC curve of the two‐miRNA panel were 0.857 (95% CI, 0813–0.901; *P* < 0.0001) and 0.845 (95% CI, 0.793–0.896; *P* < 0.0001) for NSCLC and NSCLC stage I, respectively. Furthermore, the panel was able to differentiate NSCLC from benign nodules with an AUC of 0.823 (95% CI, 0.730–0.915; *P* < 0.0001). Furthermore, logistic regression analysis revealed the two‐miRNA panel as an independent risk factor for NSCLC (OR = 16.128, *P* < 0.0001). In conclusion, miR‐520c‐3p and miR‐1274b have biomarker potential for early diagnosis of NSCLC in multiple centres.

## Graphical abstract

Sensitive and specific biomarkers for the detection of non‐small‐cell lung cancer (NSCLC) are urgently needed. Here, we investigated the diagnostic potential of extracellular vesicle (EV) miRNAs as biomarkers for NSCLC in a multiphase case–control study. We report that EV‐miR‐520c‐3p and miR‐1274b hold potential as novel diagnostic biomarkers for early‐stage NSCLC.

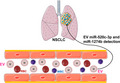

**Abbreviations**
AUC: area under of the ROC curve
Ct: quantification cycle
DEPC: diethylpyrocarbonate
EVs: Extracellular vesicles
miRNA: microRNA
MVs: microvesicles
NSCLC: non‐small‐cell lung cancer
ROC: receiver operating characteristic curves
RT‐qPCR: quantitative reverse‐transcription polymerase chain reaction
TLDA: TaqMan Low Density Array

## 1 Introduction

According to Cancer Statistics 2020, lung cancer is one of the most common cancers worldwide and the leading cause of cancer‐related deaths among males and females [1]. Non‐small‐cell lung cancer (NSCLC) accounts for 80–90% of lung cancers [2]. Early‐stage NSCLCs are asymptomatic and difficult to diagnose, and approximately 60% of NSCLC patients are diagnosed at an advanced stage with a 5‐year survival rates of only approximately 5% [3]. At present, NSCLC diagnosis mainly relies on imaging examinations, such as X‐rays, computed tomography (CT) scans and PET‐CT scans, and validated by bronchoscopy and biopsy, which are challenging tissue sampling procedures. Although low‐dose CT (LDCT) has the potential for earlier lung cancer diagnosis among high‐risk individuals, only a few people have been screened by this method [1]. Additionally, neuron‐specific enolase (NSE), squamous cell carcinoma antigen (SCC), cancer antigen 125 (CA125), carcinogen‐embryonic antigen (CEA) and cytokeratin 19 fragment (CYFRA21‐1) are commonly used as serologic tumour markers for NSCLC diagnosis and prognosis in clinical settings. Nevertheless, these indexes are limited by low sensitivity and specificity [4]. For these reasons, finding a convincing, sensitive and specific way to diagnose NSCLC remains an urgent task for clinicians and researchers.

Extracellular vesicles (EVs), cell‐derived lipid bilayer‐delimited particles, are released after fusion of endosomes with the plasma membrane (exosomes) or shed from the plasma membrane (microvesicles, MVs). EVs diameter range from 30–1000 nm, and the vast majority of EVs are smaller than 200nm. It has been demonstrated that multiple cell types can secrete EVs, which can be found in diverse body fluids [5]. In recent years, multiple studies have investigated the function of EVs in tumours. These tumour‐derived EVs can mediate communication between cells in the tumour microenvironment [7, 8].

MicroRNAs (miRNAs) are a family of small, single‐stranded noncoding RNAs that are found in diverse organisms and may play a crucial role in gene regulation [8]. It has been reported that miRNAs are related to various biological processes, such as cell death, differentiation, cell proliferation and metastasis [9]. Furthermore, multiple studies have investigated the diagnostic value of miRNAs in cancer [10]. Our group and others also demonstrated that circulating miRNAs in patients with NSCLC have unique profiles, and we identified some miRNAs that are elevated or decreased specifically in serum or plasma samples of NSCLC patients [12, 13, 14].

Recently, miRNAs carried by EVs, as opposed to free miRNAs, were demonstrated to be as more specific and excellent candidate molecular biomarkers for cancers [15, 16, 17]. It has been demonstrated that the EV miRNAs pattern of NSCLC patients is different from that of healthy people [18, 19, 20, 21, 22]. For instance, miR‐181‐5p, miR‐30a‐3p, miR‐361‐5p and miR‐30e‐3p in EVs were reported as adenocarcinoma‐specific of NSCLC patients, while miR‐15b‐5p, miR‐10b‐5p and miR‐320b in EVs were found to be squamous cell carcinoma‐specific of NSCLC patients [18]. Moreover, levels of EV‐associated let‐7f and miR‐30e‐3p are reported to be correlated with poor outcome of NSCLC patients [21]. Nevertheless, only two focused on the potential diagnostic value of circulating EV‐related miRNAs in early‐stage NSCLC, and a multicentric study of NSCLC miRNAs has not been reported. Therefore, a systematic analysis of circulating miRNAs from multicentric NSCLC cases, especially in the early stage, is needed.

In this study, we used high‐throughput TaqMan Low Density Array (TLDA) to initially identify a unique pattern of circulating EV miRNAs of NSCLC patients and then applied quantitative reverse‐transcription polymerase chain reaction PCR (RT‐qPCR) to validate the TLDA results in individual samples from several medical centres. We further estimated the value of early‐stage diagnosis and differential diagnosis of the identified miRNAs for NSCLC and their ability to evaluate the tumour dynamics.

## 2 Materials and methods

### 2.1 Participants and sample collection

We enrolled 207 participants who were diagnosed with NSCLC according to WHO criteria from three different medical centres that included Jinling Hospital, Nanjing Drum Tower Hospital and Nanjing Chest Hospital. In addition, we also collected samples from 168 age‐ and sex‐matched healthy individuals and 31 benign nodule patients from Jingling Hospital as normal and benign disease controls, respectively. A total of 3–10 mL of peripheral venous blood after 12 h of fasting was obtained from each patient before surgery and pharmacotherapy to acquire the serum or plasma samples. In brief, the blood samples of all participants were collected using a vacuum tube with EDTA‐K_2_ for preparing plasma samples, and serum was collected using a vacuum tube with separation gel for preparing serum samples. The bloods were centrifugated at 1500 g for 10 min at room temperature to remove cell fractions, and the supernatants were recentrifuged at 12 000 g for 10 min at 4 ℃ to completely remove cell debris. The final supernatants were transferred to a new tube and stored at −80 ℃ for further analysis. The project was approved by the ethics committee of each participating medical centre was in accordance with the Declaration of Helsinki. Each participant that joined this study provided informed consent.

### 2.2 Study design

We first enrolled 36 participants who were diagnosed with NSCLC and 36 age‐ and sex‐matched healthy individuals from Jinling hospital, and every 12 samples in each group were mixed as a pool. The high‐throughput TaqMan Low Density Array (TLDA) was then applied to scan the unique pattern of EV miRNAs of NSCLC in the 6 pooled plasma samples (Table S1). We subsequently confirmed the results of TLDA by individual RT‐qPCR assays of 38 serum and 38 corresponding plasma samples (from 19 patients and 19 controls) from Jinling Hospital. Furthermore, we examined the significantly increased serum miRNAs in three independent cohorts from Jinling Hospital (training set with 41 NSCLC patients and 41 controls) and two other medical centres, including Nanjing Drum Tower Hospital (validation set with 30 NSCLC patients and 29 controls) and Nanjing Chest Hospital (testing set with 31 NSCLC patients and 31 controls). In addition, we also investigated the changes of the identified miRNAs in 38 NSCLC patients before and after surgery (from Jinling Hospital). Detailed clinical information of patients involved in RT‐qPCR validation is shown in Table 1.

**Table 1:** Demographic and clinical features of the NSCLC patients and controls of the three hospitals cohorts

Variable	Jinling Hospital			Drum Tower Hospital		Nanjing Chest Hospital	
	Normal control	NSCLC	Benign nodules	Normal control	NSCLC	Normal control	NSCLC
Number	*n* = 60	*n* = 98	*n* = 31	*n* = 29	*n* = 30	*n* = 31	*n* = 31
Age	55.3 ± 7.6	60.3 ± 11.2	52.7 ± 12.7	57.2 ± 7.8	60.7 ± 7.3	50.2 ± 5.7	58.6 ± 9.3
≤59	52	37	17	21	18	28	17
>59	8	61	14	8	12	3	14
Sex							
Male	33	54	21	17	19	14	18
Female	27	44	10	12	11	17	13
Smoking status							
Ever and current	22	41	14	7	10	8	20
Never	38	57	17	22	20	23	11
Tumour subtype							
AC	–	82	–	–	25	–	25
SCC	–	16	–	–	5	–	6
Tumour stage							
I	–	61	–	–	19	–	16
II	–	13	–	–	5	–	5
III	–	19	–	–	5	–	8
IV	–	5	–	–	1	–	0
Unknown	–	0	–	–	0	–	2

The data are expressed as the mean (SD).

### 2.3 TLDA of circulating EV miRNAs

The TLDA was designed for two‐step RT‐qPCR. In the reverse‐transcription step, total RNA was reverse transcribed to cDNA using random primers from a high capacity CM Archive kit and performed according to operating manual. All steps were performed by the 7900 HT Fast Real‐Time PCR System (Applied Biosystems, Foster City, CA, USA). The results are shown as Ct and normalized to the reference gene according to the manufacturer’s recommendations. The relative level was calculated using the comparative Ct method (2^−ΔCt^).

### 2.4 Serum and plasma EVs isolation

For TLDA analysis, EVs were isolated by ultracentrifugation (UC) from pooled plasma samples. The UC method was performed according to previously described methods [22]. For the RT‐qPCR assay, EVs of plasma or serum were isolated by ExoQuick solution (System Biosciences Inc., Mountain View, CA, USA). According to the manufacturer’s instruction, 250 μL of serum or plasma was transferred to a new sterile tube and then mixed with 63 μL of EV precipitation solution. The acquired EVs were used for further analysis.

### 2.5 Transmission electron microscopy

The EVs were isolated from pooled plasma and serum samples using ExoQuick solution, resuspended with 1 × PBS, dropped on a cooper mesh and incubated at room temperature for 20 min. Excess liquid at the edge was cleaned. Sequentially, 2% phosphotungstic acid solution was added, negative staining was performed at room temperature for 10 min, and the copper plate was air‐dried. The microphotographs were acquired by using a JEM‐1011 transmission electron microscope.

### 2.6 Western blot and NanoSight

EV proteins were lysed with RIPA buffer and separated on a 10% SDS/PAGE gel followed by transfer onto a PVDF membrane. The membrane was incubated with the following antibodies: anti‐CD 9, 1 : 1000 diluted, abs102483, Absin; anti‐TSG101, 1 : 5000 diluted, DF8427, Affinity bioscience; anti‐CD 63, 1 : 1000 diluted, Abs110250, Absin; anti‐Alix, 1 : 1000 diluted, DF9027, Affinity bioscience; and anti‐ALB, 1 : 1000 diluted, DF6396, Affinity bioscience. Subsequently, the membrane was rinsed four times in Tris‐buffered saline containing 0.5% Tween‐20 (TBST) and incubated with secondary antibody (Anti‐rabbit IgG, HRP‐linked Antibody, 1: 3000 diluted, 7074, CST). We detected the proteins with ECL western blotting substrate (Thermo Fisher Scientific, Waltham, MA, USA). We used the NS300 equipment (NanoSight, Malvern Panalytical, Worcestershire, UK) to analyse the size distribution of EVs. Videos were recorded at camera level 9 with the minimal expected particle size, minimum track length, and blur setting, all set to automatic. Every sample for NanoSight was diluted with PBS (1 : 1000, Gibco, Waltham, NY, USA), and 1000 μL of each sample was added to the chamber under the video recording.

### 2.7 RNA extraction and RT‐qPCR assays

EVs total RNA was extracted with the TRIzol Reagent (Invitrogen, MA, USA) according to our previous report [23]. A hydrolysis probe‐based RT‐qPCR assay of miRNAs was performed as previously reported [23]. Relative quantitation was used, and the levels of miRNAs in serum or plasma samples were normalized to the level of reference cel‐miR‐39 (a synthetic miRNA 5’‐UCACCGGGUGUAAAUCAGCUUG‐3’, GenePharma) by 2^–Δct^, where ΔCt = ΔCt_target miRNA _– Ct_cel‐miR‐39_.

### 2.8 Evaluation of miRNA levels in EVs and EV‐free fraction by RT‐qPCR assays

To determine whether the significantly altered miRNAs are primarily encapsulated in EVs or circulating freely in circulation, EVs and corresponding EV‐free fractions were separated from individual samples of additional 12 NSCLC patients and 12 controls using UC method according to previously described [22]. MiRNAs in EVs and EV‐free were extracted and purified using Trizol Reagent (Invitrogen, Carlsbad, CA), per manufacturer’s instructions with minor modification as described [24]. The absolute concentrations of miRNA in EVs and EV‐free samples were assessed by RT‐qPCR assays as previously described [24].

### 2.9 Statistical analysis

Statistical analysis was performed by SPSS 24.0 statistical software and graphpad prism 7.0 (Graphpad, San Diego, CA, USA). The nonparametric Mann‐Whitney U‐test was used to assess the differences between the groups, and the differences for other variables were calculated by Student's t‐test or two‐sided χ^2^ test. A *P*‐value < 0.05 was considered to be statistically significant. For our two‐miRNA signature, the risk score for each patient was calculated as follows:

Risk score = (4.553 × expression level of miR‐520c‐3p) + (0.422 × expression level of miR‐1274b). The ROC curves were generated to evaluate the accuracy of miRNA predication for NSCLC. Finally, univariate and multivariate logistic regression analyses were carried out to assess the predictive power of the candidate miRNAs for NSCLC.

### 2.10 Target gene prediction and GO/KEGG pathway enrichment analysis

For each miRNA with differential expression between NSCLC and normal controls, its potential target genes that were predicted by miRanda [25] (http://www.microrna.org/microrna/home.do) were included for further analysis. Afterward, we performed Gene Ontology (GO) annotation and Kyoto Encyclopedia of Genes and Genomes (KEGG) pathway analyses using the cluster Profiler package of R software. *P*‐value < 0.05 was set as the cut‐off criterion.

## 3 Results

### 3.1 Characterization of serum and plasma EVs

**Fig. 1:**
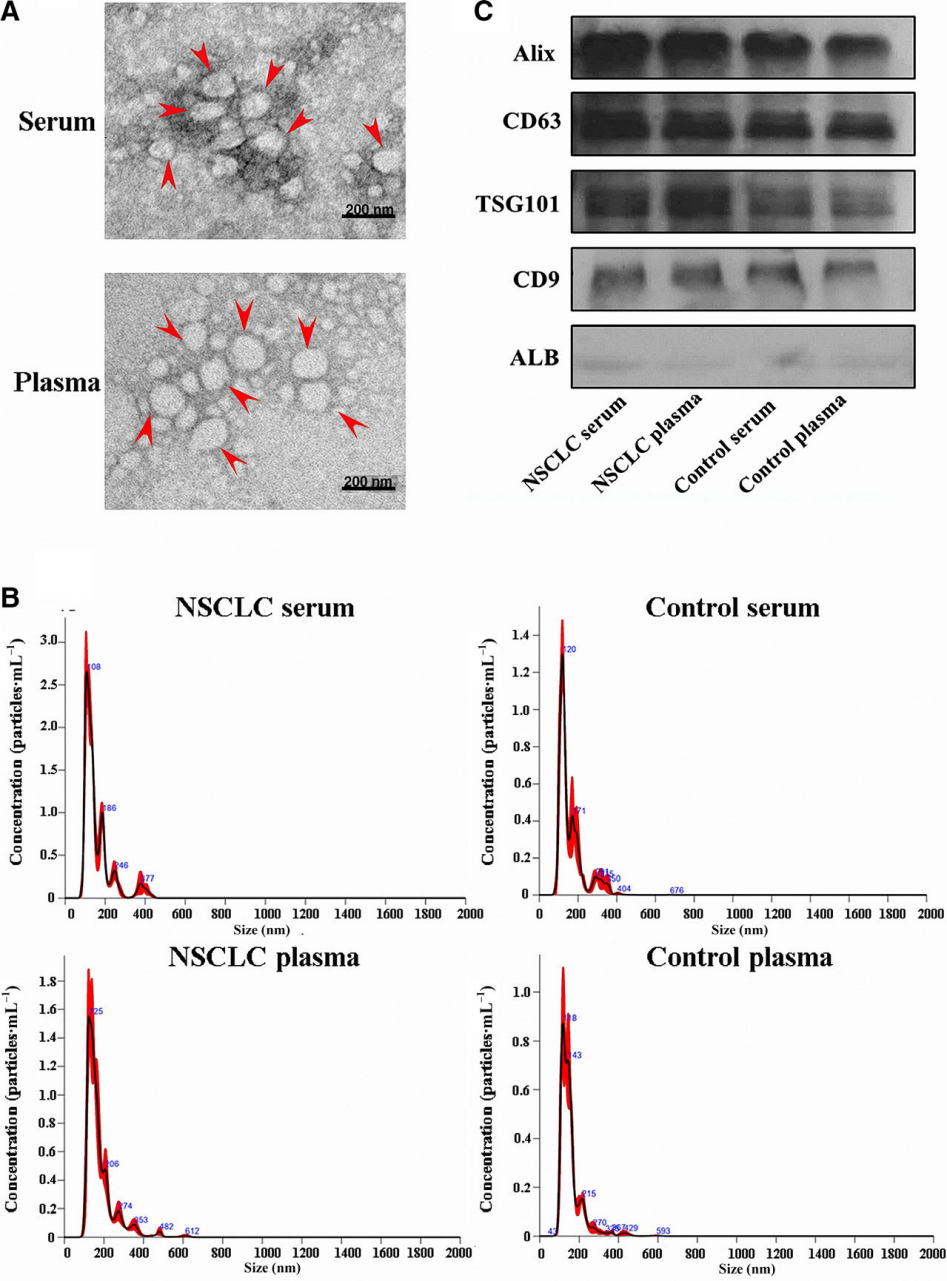
Characterization of EVs derived from the serum and plasma of NSCLC patients and controls. (A) The shape and structure of serum and plasma EVs isolated by EXOquick kit under TEM. The red arrow represents EVs with typical characteristics (scale bars are 200 nm). (B) The size of EVs derived from control groups and NSCLC groups was analysed by NTA. (C) Western blots of EVs membrane markers, including Alix, CD63, TSG101, CD9 and one negative marker ALB.

Because the quality of the EVs separation is a prerequisite for the accurate analysis of EV miRNAs, we used three different techniques to characterize the EVs that were isolated from serum and plasma by the ExoQuick kit. We visualized EVs by transmission electron microscopy (TEM) to observe the typical morphologies of EVs (Fig. 1A). Then, we measured the size distribution and concentrations of the EVs by NanoSight. The EVs were approximately 50–150 nm in size (Fig. 1B). Finally, we performed western blotting to confirm the presence of the classic markers of Alix, CD63, TSG101, CD9 and one negative marker ALB (Fig. 1C). All the results demonstrated that we successfully isolated EVs from serum and plasma.

### 3.2 Analysis of plasma EV miRNA profiles by TLDA

**Fig. 2:**
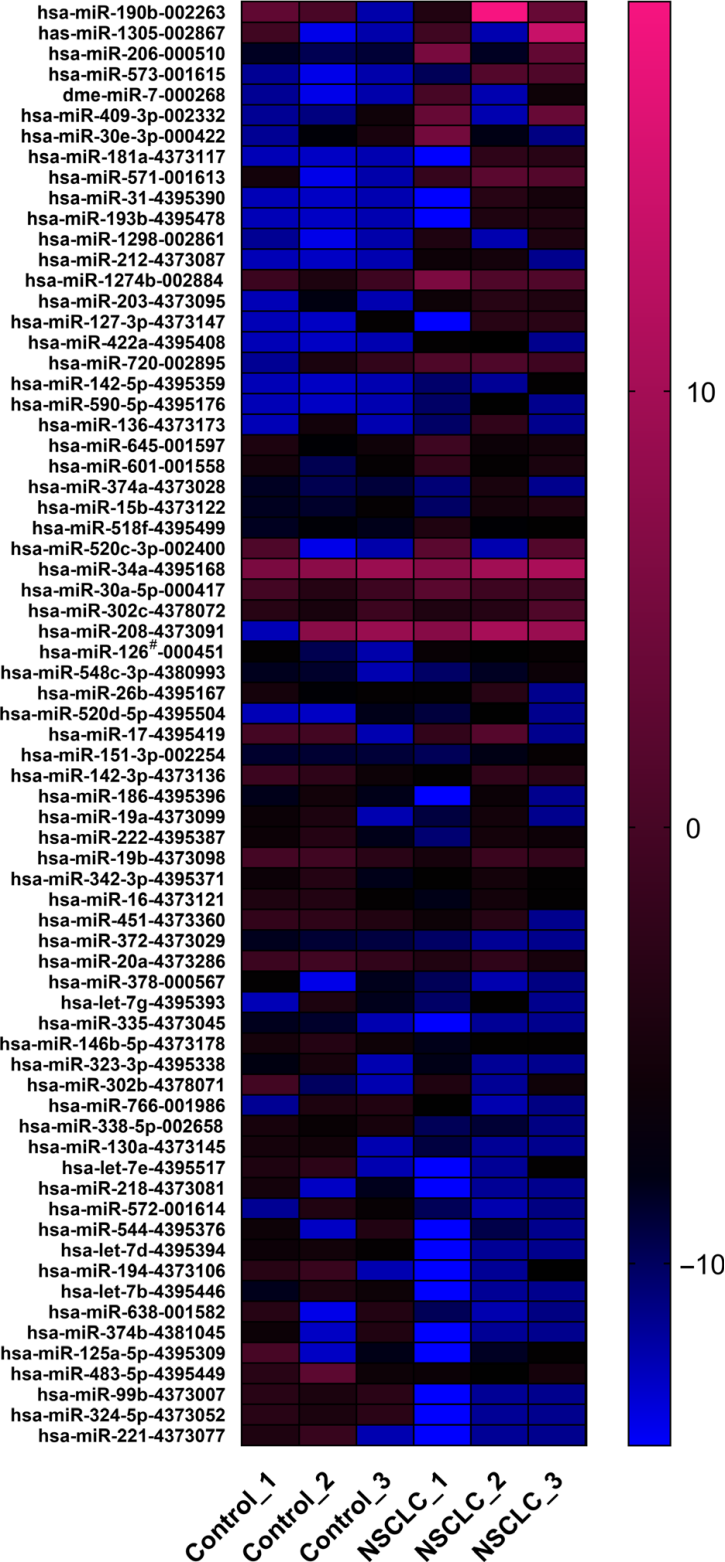
Heat map describing the markedly upregulated EV miRNAs from TLDA. EVs were derived from 3 pooled serum samples of NSCLC patients and 3 pooled serum samples of controls. Each pool was consisted of 12 individual samples. Heatmap of pairwise correlation values of the 6 samples. 37 miRNAs were upregulated and 33 were downregulated for more than 2‐fold.

TLDA was used to initially measure 745 miRNAs in 6 pools. There was no significant difference in age or sex distribution between the different groups (Table S1). A heat map visualized significant differences in miRNA profiles between the NSCLC and healthy control groups (Fig. 2). We considered a miRNA to be notably upregulated if its average Ct was < 35 in at least two pools of NSCLC, and the concentration had a ≥ twofold increase in the NSCLC groups in comparison with the normal groups. According to this criterion, 37 miRNAs were regarded as upregulated and 33 were downregulated (Fig. 2).

### 3.3 Validation of the TLDA results by RT‐qPCR

**Fig. 3:**
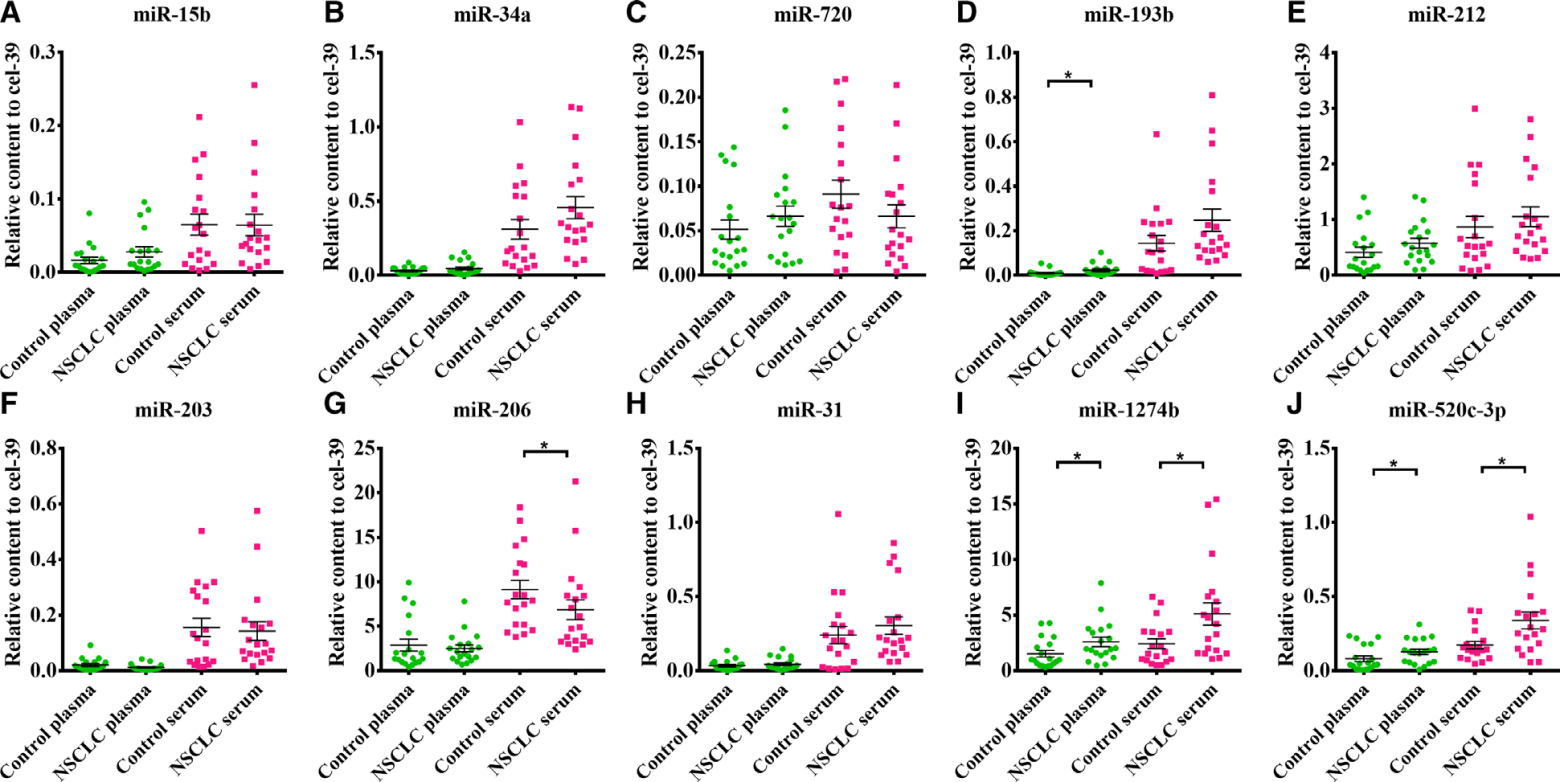
qPCR data on miRNA expression levels of selected miRNAs of serum and plasma in the screening set(n_NSCLC_ = 19, n_control_ = 19, A–J. The expression of miR‐15b (A), miR‐34a (B), miR‐720 (C), miR‐193b (D), miR‐212 (E), miR‐203 (F), miR‐206 (G), miR‐31 (H), miR‐1274b (I) and miR‐520c‐3p (J) was normalized by cel‐miR‐39. miR‐520c‐3p and miR‐1274b uniformly consistently elevated in EVs from both plasma and serum. Horizontal bars represent the mean value. p‐values were measured by Mann–Whitney independent t‐test. *P < 0.05.

Of the 37 upregulated miRNAs, we selected those elevated by at least threefold in the NSCLC groups to validate. Furthermore, newly identified miRNAs, miRNA‐star miRNAs and nonconserved miRNAs were also excluded for further investigation (Table S2). According to these criteria, 13 miRNAs were identified and selected for individual validation in the screening set that included 19 NSCLC cases and 19 controls from Jinling Hospital. We examined the 13 miRNAs in both plasma and serum samples. Of the 13 miRNAs tested, three miRNAs miR‐7, miR‐181a and miR‐573 were undetectable; ten were detectable in either plasma or serum EVs, but only miR‐520c‐3p and miR‐1274b showed uniformly significant increases in EVs from both plasma and serum (*P* < 0.05); and the two miRNAs were chosen for further study (Fig. 3). In addition, by comparison, we found that miRNAs in EVs from serum had higher levels than that from plasma (Fig. 3). Therefore, we chose serum as the candidate specimen type for the following experiments.

### 3.4 Validation of miR‐520c‐3p and miR‐1274b in serum samples from three different medical centres

**Fig. 4:**
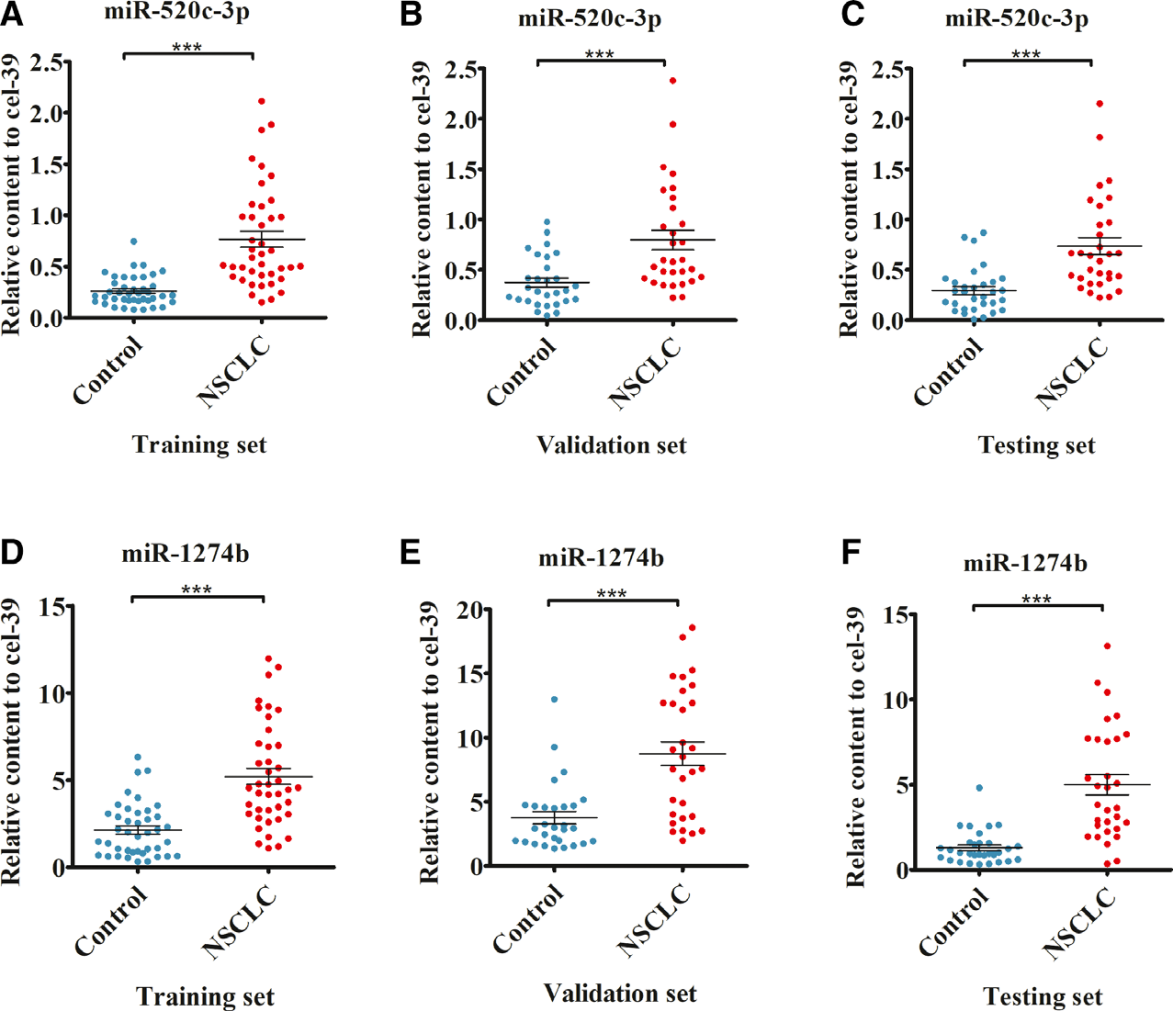
Expression levels of miR‐520c‐3p and miR‐1274b in the three sets from different hospitals (A–F). The relative expression of miR‐520c‐3p and miR‐1274b in training set (*n*_NSCLC_ = 41, *n*_control_ = 41), validation set (*n*_NSCLC_ = 30, *n*_control_ = 29) and testing set (*n*_NSCLC_ = 31, *n*_control_ = 31) calculated by Delta Ct method. Horizontal bars represent the mean. The two miRNAs in the three independent sets were uniformly significantly increased in the NSCLC patients. *P*‐values were measured by Mann–Whitney independent t‐test. ***P < 0.001.

miR‐520c‐3p and miR‐1274b were further validated in serum of NSCLC patients from three different medical centres that included Jinling Hospital (training set), Nanjing Drum Tower Hospital (validation set) and Nanjing Chest Hospital (testing set). Consistent with the results of the screening set, the levels of miR‐520c‐3p and miR‐1274b in the three independent sets were consistently significantly elevated in the NSCLC patients in comparison with the healthy controls (*P* < 0.001, Fig. 4).

### 3.5 Comparison of miR‐520c‐3p and miR‐1274b between NSCLC and benign nodules

**Fig. 5:**
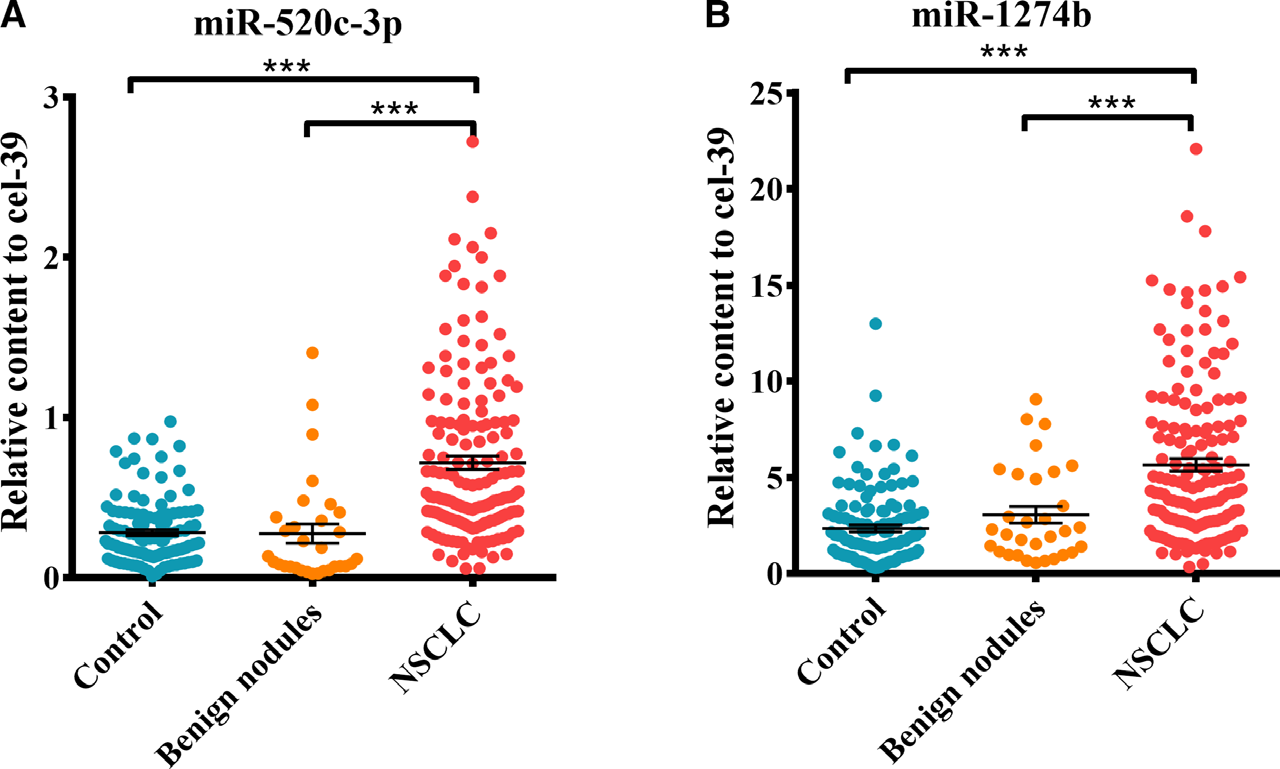
Expression levels of miR‐520c‐3p (A) and miR‐1274b (B) between normal controls, benign nodules patients and NSCLC patients. The relative expression of miR‐520c‐3p (A) and miR‐1274b (B) was detected in healthy groups (*n* = 120), benign nodules patients (*n* = 31) and NSCLC patients (*n* = 159). The level of two miRNAs was elevated significantly in NSCLC patients, but there is no difference between healthy groups and benign nodules patients. *P*‐values were measured by Mann–Whitney independent *t*‐test. ***P < 0.001.

Furthermore, we analysed miR‐520c‐3p and miR‐1274b in serum from patients with benign nodules (*n* = 31). We observed no significant differences in the levels of the two miRNAs between benign nodule patients and healthy controls (Table S3), but miR‐520c‐3p and miR‐1274b were markedly lower than those in NSCLC patients (*P* < 0.001 and *P* < 0.01) (Fig. 5).

### 3.6 The levels of miR‐520c‐3p and miR‐1274b in different stages and subtypes

We then analysed the levels of miR‐520c‐3p and miR‐1274b in different pathological stages and histological subtypes of NSCLC. The levels of miR‐520c‐3p and miR‐1274b were significantly increased in clinical stage I NSCLC patients, and the mean fold change of miR‐520c‐3p and miR‐1274b was 2.5 and 2.2 in comparison with the healthy group. In addition, the levels of the two miRNAs were also obviously increased in other stages, but there was no significant difference among the different stages (Table S4). Furthermore, when compared with the healthy group and benign nodules, the mean fold change of miR‐520c‐3p was 2.5 and 2.7 in NSCLC adenocarcinoma (AC) patients, 2.9 and 2.9 in NSCLC squamous carcinoma (SCC) patients, and the mean fold change of miR‐1274b was 2.3 and 1.7 in AC patients and 2.1 and 1.6 in SCC patients. Nevertheless, there was no significant difference of the two miRNAs between AC patients and SCC patients (Table S3).

### 3.7 Prediction of NSCLC cases by ROC curve analysis

Subsequently, we generated a ROC curve to analyse the diagnostic value of miR‐520c‐3p and miR‐1274b for NSCLC in different patient sets. The areas under the curve (AUCs) of the two miRNAs ranged from 0.790 to 0.895 in the above sets. Additionally, the AUCs of miR‐520c‐3p and miR‐1274b were 0.819 (95% CI, 0.770–0.868) and 0.788 (95% CI, 0.736–0.880) when all sets were combined (Tables S5 and S6).

**Fig. 6:**
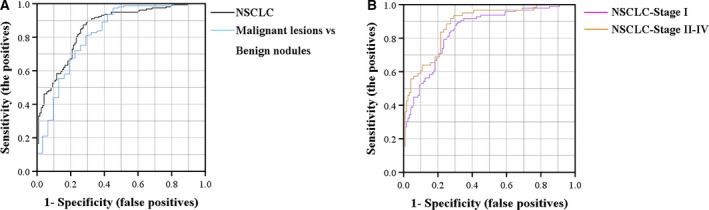
Diagnostic values of miRNAs by qPCR. The ROC and AUC were used to determine the sensitivity and specificity of select miRNAs. (A) ROC curve for the two‐miRNA panel to distinguish 159 NSCLC cases from 120 normal controls in all sets (black line, AUC = 0.857), and ROC curve for the two‐miRNA panel to distinguish 159 malignant lesion patients from 31 benign nodule patients (blue line, AUC = 0.823). (B) ROC curve for the two‐miRNA panel to distinguish 96 clinical stage I cases from 120 controls in all sets (purple line, AUC = 0.845) and ROC curve for the two‐miRNA panel to distinguish 61 stage II–IV cases from 120 controls (orange line, AUC = 0.881).

To evaluate the diagnostic value of the two‐miRNA panel, we calculated a risk score and used it to predict NSCLC case and control status. Because early‐stage NSCLC patients can undergo a complete tumorectomy and early detection of this cancer will greatly prolong greatly, therefore, we investigated the diagnostic values of the miR‐panel for stage I and stages Ⅱ‐Ⅳ in all cases. The AUCs for stage I and stage II‐IV were observed to be 0.845 (95% CI, 0.793–0.829) and 0.881 (95% CI, 0.831–0.932), respectively (Table S6). When the optimal cut‐off value was 2.69, the diagnostic sensitivity of the panel for stage I NSCLC was 82.3% and the specificity was 73.9% (Fig. 6B). Furthermore, the AUC of the two‐miRNA panel differentiating NSCLC from benign nodules was 0.823 (95% CI, 0.730–0.915) (Fig. 6A).

### 3.8 Changes in miR‐520c‐3p and miR‐1274b in NSCLC patients after tumorectomy

**Fig. 7:**
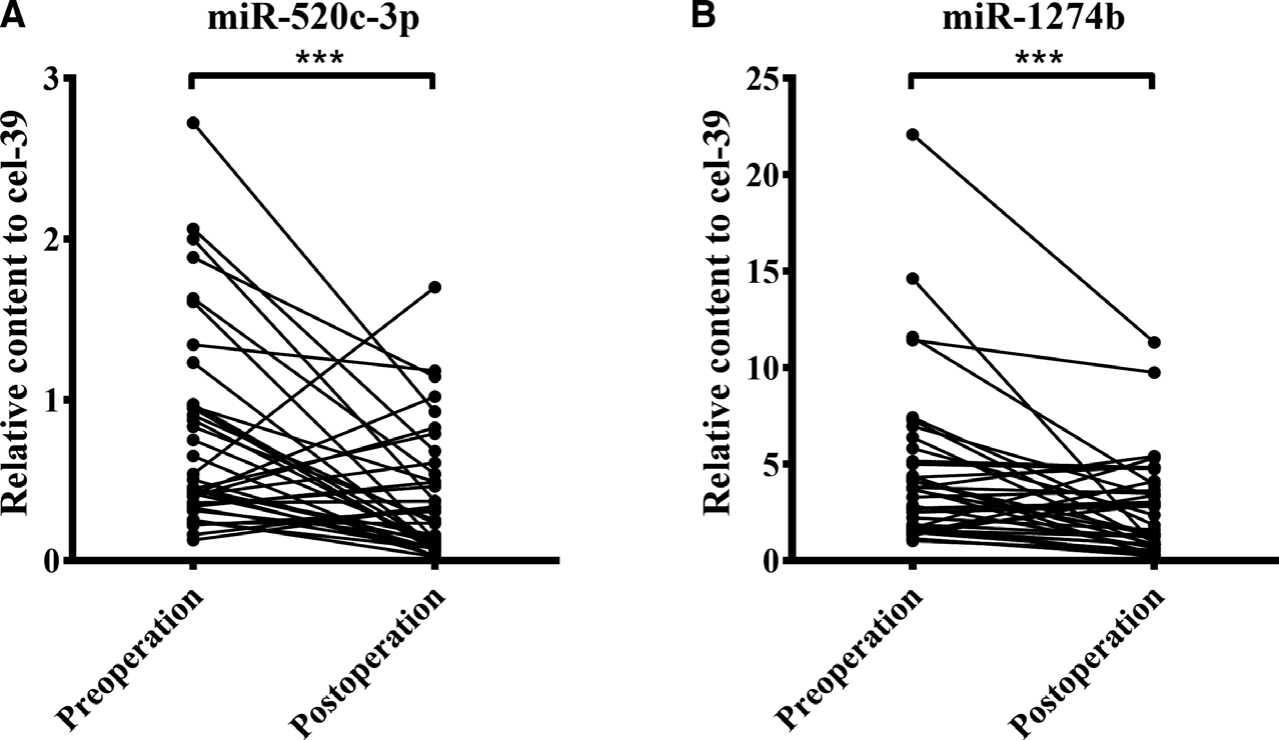
Expression levels of miR‐520c‐3p and miR‐1274b before and after operations in 38 NSCLC patients. The relative expression of miR‐520c‐3p (A) and miR‐1274b (B) were tested before and after operations in NSCLC patients (*n* = 38). The expression levels of the two miRNAs were markedly decreased after surgery. *P*‐values were measured by paired *t*‐test. ***P < 0.001.

Whether miR‐520c‐3p and miR‐1274b in EVs could dynamically monitor the progression of NSCLC is also an important factor to weight their clinical value. Therefore, we compared the levels of miR‐520c‐3p and miR‐1274b in 38 NSCLC cases before and 7 days after tumorectomy. We observed that the levels of the two miRNAs were markedly decreased after surgery (*P* < 0.001) (Fig. 7).

### 3.9 Logistic regression analysis of miR‐520c‐3p and miR‐1274b

We conducted a univariate logistic regression analysis to investigate the risk prediction value of the two miRNAs for NSCLC. The odds ratios (ORs) of miR‐520c‐3p, miR‐1274b and the two‐miRNA panel were 10.048 (95% CI, 4.153–24.432), 9.771 (95% CI, 4.036–23.655) and 16.128 (95% CI, 6.702–38.813), respectively. Then, a multivariate logistic regression analysis was used to investigate the association between the levels of the two miRNAs and clinical characteristics of NSCLC patients. miR‐520c‐3p, miR‐1274b and the two‐miRNA panel were independently associated with NSCLC after adjusting for gender, age and smoking status (Table S7).

### 3.10 Majority of serum miR‐520c‐3p and miR‐1274b are encapsulated in EVs

**Fig. 8:**
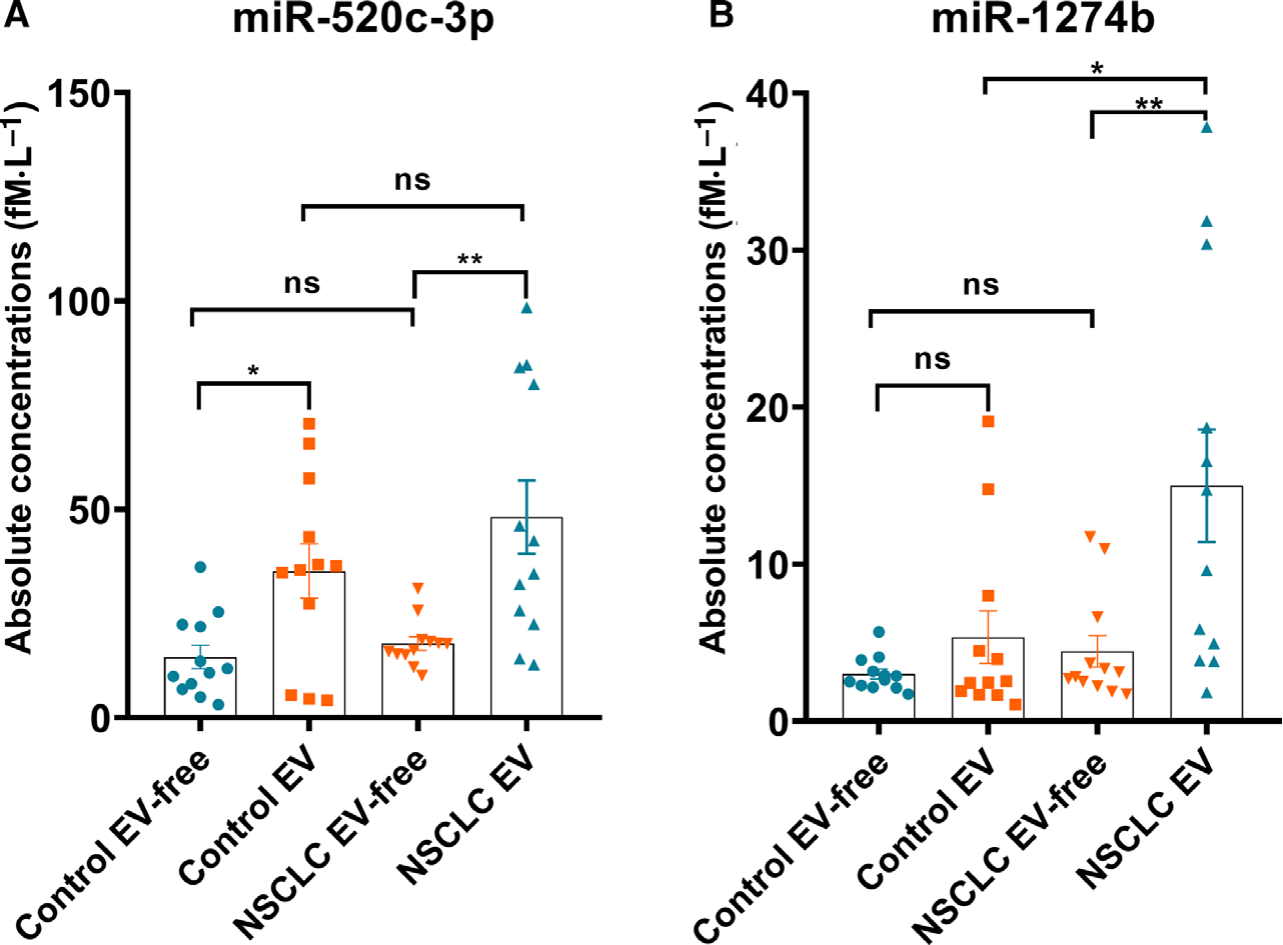
Expression levels of miR‐520c‐3p and miR‐1274b in serum EVs and EV‐free fraction between normal controls and NSCLC patients. The concentrations of miR‐520c‐3p and miR‐1274b in serum EVs and EV‐free fraction were detected in healthy groups (*n* = 12), and NSCLC patients (*n* = 12). EV and EV‐free fraction samples were obtained by ultracentrifugation. *P*‐values were measured by Mann–Whitney independent *t*‐test. *P < 0.05, **P < 0.01.

To determine whether miR‐520c‐3p and miR‐1274b are encapsulated inside of EVs or are freely circulating in serum, the concentrations of these two miRNAs in both EVs and EV‐free samples of individual participant from additional 12 NSCLC patients and 12 age sex‐matched controls were then determined by RT‐qPCR. The demographic and clinical of these patients and controls for EVs and EV‐free fractions separation is shown in Table S8. Consequently, we found that the mean levels of miR‐520c‐3p were markedly higher in serum EV than EV‐free samples in both NSCLC group and control group (Fig. 8A). Moreover, the levels of miR‐1274b were also higher in EV than EV‐free serum in both NSCLC group and control group (Fig. 8B). Furthermore, the miR‐1274b levels in EV samples were still significant increased in NSCLC patients when compared with controls (Fig. 8B). Together, these results can partially prove that miR‐520c‐3p and miR‐1274b were mainly located in EVs.

### 3.11 Target analysis of miR‐520c‐3p and miR‐1274b by KEGG/GO

**Fig. 9:**
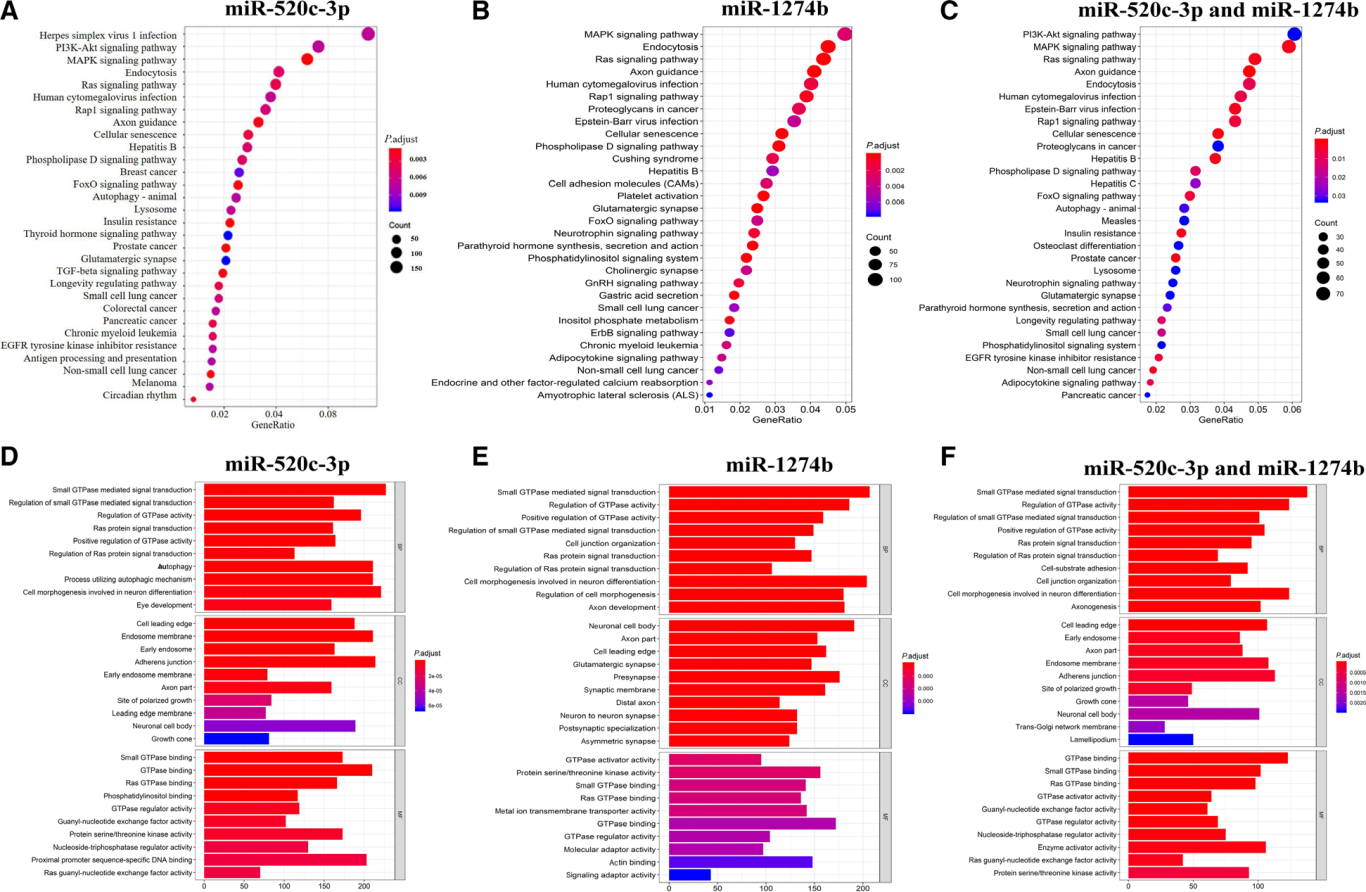
GO/KEGG enrichment analysis result with a fold change > 2. GO/KEGG enrichment of mRNAs targeted by the two miRNAs. BP biological process, CC cellular component, MF molecular function. (A) A bubble plot of enriched KEGG pathways (target genes of miR‐520c‐3p). (B) A bubble plot of enriched KEGG pathways (target genes of miR‐1274b). (C) A bubble plot of KEGG pathways (co‐target genes of miR‐520c‐3p and miR‐1274b). (D) A bar plot of GO terms (target genes of miR‐520c‐3p). (E) A bar plot of GO terms (target genes of miR‐1274b). (F) A bar plot of GO terms (co‐target genes of miR‐520c‐3p and miR‐1274b).

We used miRanda [25] to predict the possible target genes of miR‐520c‐3p and miR‐1274b. The miRanda algorithm predicted that miR‐520c‐3p could target 7550 genes, miR‐1274b could target 6845 genes, and the two miRNAs co‐targeted 3647 genes. We used KEGG enrichment of mRNAs predicted to be targeted by miR‐520c‐3p and miR‐1274b to reveal the potential function of the two miRNAs (Fig. 9A,B). Then, we analysed the two miRNA co‐targeted genes, which were enriched in pathways in cancer, such as the PI3K‐Akt signalling pathway, MAPK signalling pathway and Ras signalling pathway (Fig. 9C). GO term enrichment analysis results varied from GO classification (Fig. 9D,E). Regarding biological processes, the co‐targeted genes were significantly enriched in small GTPase‐mediated signal transduction and regulation of GTPase activity. For the cellular components, the co‐targeted genes were significantly enriched in cell leading edge and endosome (Fig. 9F). Regarding the molecular function, the co‐targeted genes were enriched in GTPase binding, small GTPase binding, Ras GTPase binding and GTPase activator activity.

## 4 Discussion

Poor early detection is a major obstacle to improving the low 5‐year survival rates of patients with lung cancer. Recently, EV miRNAs have been intensively demonstrated as potential biomarkers to diagnose tumorigenesis or evaluate prognosis [14], and EV miRNA signatures have been reported in NSCLC [18, 22]. Multicentric and repeatable validation studies are essential for achieving true translation relevance and advancing EV miRNAs from the bench to clinical practice. In this study, we performed a systematic analysis of circulating EV miRNA profiling by TLDA and then conducted repeated validation of candidate miRNAs in NSCLC patients from three different hospitals by RT‐qPCR. We found that the levels of miR‐520c‐3p and miR‐1274b were significantly and steadily increased in stage I NSCLC patients in comparison with normal controls. The two‐miRNA panel exhibited high diagnostic accuracy for early‐stage cases. Moreover, miR‐520c‐3p and miR‐1274b could accurately discriminate NSCLC from benign nodules. Additionally, we observed that the levels of the two miRNAs obviously decreased after tumorectomy. Furthermore, forward stepwise logistic regression analysis showed that the ORs of the two miRNAs and their panel were higher than 9.97, which suggests that the two miRNAs are independent risk factors for NSCLC. Our results revealed that miR‐520c‐3p and miR‐1274b have potential to be auxiliary diagnostic tools for then of NSCLC.

Some studies have detected ‘free‐circulating’ miRNAs in NSCLC patients [12, 13, 27, 28]. Nevertheless, miR‐520c‐3p and miR‐1274b have not been previously identified in ‘free‐circulating’ studies of lung cancer. This is in line with the view that EVs undergo a unique pathway for the release of nucleic acids from cells and contain selectively packaged miRNA species, which suggests that EV miRNAs may be more specific for disease diagnosis [28]. Moreover, the correlation between the two miRNAs and prognosis needs to be investigated further. Finally, longitudinal studies should be pursued to assess whether miR‐520c‐3p and miR‐1274b can detect time‐critical NSCLC recurrences.

It has been reported that the expression level of miR‐520c‐3p in nontumour tissues is higher than that in lung adenocarcinoma tissues. miR‐520c‐3p may act as a tumour suppressor to regulate various biological processes [30, 31, 32]. For instance, miR‐520c‐3p inhibits the invasion and migration of breast cancer by targeting IL‐8 and suppresses the invasion and migration of lung cancer by targeting AKT1 and AKT2 [30, 33]. miR‐1274b was found to be upregulated in human lung adenocarcinoma side population cells, which is the root cause of tumour growth and development [33]. Nonetheless, the function of miR‐1274b has not been clearly elucidated. In this study, we used a bioinformatics method to predict the biological function of miR‐1274b and found that it was involved in multiple signalling pathways, such as the Ras signalling pathway, MAPK signalling pathway and PI3K‐Akt signalling pathway, which are associated with tumorigenesis and development.

Adequate specimen type is an essential prerequisite for accurate test results. In this study, we compared the levels of the selected miRNAs between plasma and serum in 38 individuals. We found that the miRNA contents were higher in serum than in plasma, which is consistent with previously reported results [23]. Therefore, we chose serum as the specimen type for further research because a higher content of miRNAs leads to higher accuracy and less interference during testing. The reason why EV miRNAs in serum are higher than in plasma is not clear. One possible reason is that platelets release more extracellular vesicles in serum than in plasma during clot formation, and the concentration of platelet microparticles in serum is far higher than that in plasma [34].

EVs isolation methods have been extensively explored in different biological fluids, such as plasma, bronchoalveolar lavage and urine samples. Modified EVs isolation techniques have been applied in RNA analysis and proteomics analysis [22]. Ultracentrifugation, ultrafiltration by nanomembranes, sedimentation by polymers and immunoaffinity capture are common methods used for EVs isolation. For plasma and serum, ultracentrifugation is the classic isolation method but is time‐consuming and requires a large sample volume. The capture of immunoaffinity EVs relies on the efficiency of the antibody and is currently limited in the laboratory [35]. The other two methods are commercialized, but sedimentation by polymers has more EVs yield than ultrafiltration by nanomembranes. For these reasons, sedimentation by polymers is a better method for clinical application. In our previous study, we compared several of the most commonly used commercial EVs isolation kits and found that they could successfully extract EVs from plasma and serum, and ExoQuick solution (SBI) had the highest efficiency of EVs extraction and low albumin impurities [23]. Therefore, in this study, SBI was used to extract EVs from blood samples.

## 5 Conclusions

In summary, we demonstrated that circulating miR‐520c‐3p and miR‐1274b were elevated in NSCLC patients compared to healthy people and benign nodule patients, and the levels of the two miRNAs obviously decreased after tumour resection. The two miRNAs may be promising and effective biomarkers for the development of precise tools for early NSCLC diagnosis.

## Conflict of interest

The authors declare no conflict of interest.

## Author contributions

CW, CZ and JL designed the study. YZ, DX, BY, WZ, YS and XW contributed to data collection. YZ, CW, JW and PL analysed the data. YZ, CW and CZ wrote the manuscript. All authors reviewed the report and approved the final version.

### Peer Review

The peer review history for this article is available at https://publons.com/publon/10.1002/1878‐0261.12889.

## Supporting information

**Table S1.** Demographic and clinical features of the NSCLC patients and controls of the TLDA samples*.
**Table S2.** Upregulated EV miRNAs in NSCLC pooled serum sample compared to normal control sample determined by TaqMan Low Density Assay.
**Table S3.** Average expression level of the individual miRNA in different subtype of NSCLC and benign nodules*.
**Table S4.** Average expression level of the individual miRNA in different NSCLC stage and the differences between stages*.
**Table S5.** Areas under the curve and the asymptotic 95% confidence intervals of the individual miRNA, the panel of two‐miRNA in the different sets.
**Table S6.** Areas under the curve and the asymptotic 95% confidence intervals of the individual miRNA, the panel of two‐miRNA in the different groups.
**Table S7.** Univariate and multivariate logistic regression analyses of parameters associated with NSCLC in all NSCLC Cases*.
**Table S8.** Demographic and clinical features of the NSCLC patients and controls for evaluation of miRNA levels in EVs and EV‐free fraction*.

## Data Availability

The datasets used and analysed during the current study are available from the corresponding author on reasonable request.

## References

[1] Siegel RL, Miller KD & Jemal A. Cancer statistics, 2020. CA Cancer J Clin 70, 7–30. 31912902 10.3322/caac.21590

[2] Planchard D, Popat S, Kerr K, Novello S, Smit EF, Faivre‐Finn C, Mok TS, Reck M, Van Schil PE, Hellmann MD*et al*. (2019) Metastatic non‐small cell lung cancer: ESMO Clinical Practice Guidelines for diagnosis, treatment and follow‐up. Ann Oncol 30, 863–870. 31987360 10.1093/annonc/mdy474

[3] Siegel RL, Miller KD & Jemal A (2018) Cancer statistics, 2018. CA Cancer J Clin 68, 7–30. 29313949 10.3322/caac.21442

[4] Schneider J (2006) Tumor markers in detection of lung cancer. Adv Clin Chem 42, 1–41. 17131623 10.1016/s0065-2423(06)42001-1

[5] Akers JC, Gonda D, Kim R, Carter BS & Chen CC (2013) Biogenesis of extracellular vesicles (EV): exosomes, microvesicles, retrovirus‐like vesicles, and apoptotic bodies. J Neurooncol 113, 1–11. 23456661 10.1007/s11060-013-1084-8 PMC5533094

[6] Reiners KS, Dassler J & Coch C (2014) Role of exosomes released by dendritic cells and/or by tumor targets: regulation of NK cell plasticity. Front Immunol 5, 91, 1–5. 24639679 10.3389/fimmu.2014.00091 PMC3945280

[7] Maacha S, Bhat AA, Jimenez L, Raza A, Haris M, Uddin S & Grivel J‐C (2019) Extracellular vesicles‐mediated intercellular communication: roles in the tumor microenvironment and anti‐cancer drug resistance. Mol Cancer 18, 55. 30925923 10.1186/s12943-019-0965-7 PMC6441157

[8] Tkach M & Théry C (2016) Communication by extracellular vesicles: Where we are and where we need to go. Cell 164, 1226–1232. 26967288 10.1016/j.cell.2016.01.043

[9] Lagos‐Quintana M, Rauhut R, Lendeckel W & Tuschl T (2001) Identification of novel genes coding for small expressed RNAs. Science 294, 853–858. 11679670 10.1126/science.1064921

[10] O'Brien J, Hayder H, Zayed Y & Peng C (2018) Overview of MicroRNA biogenesis, mechanisms of actions, and circulation. Frontiers in Endocrinology 9, 402. 30123182 10.3389/fendo.2018.00402 PMC6085463

[11] Farazi TA, Spitzer JI, Morozov P & Tuschl T (2011) miRNAs in human cancer. J Pathol 223, 102–115. 21125669 10.1002/path.2806 PMC3069496

[12] Chen X, Hu Z, Wang W, Ba Y, Ma L, Zhang C, Wang C, Ren Z, Zhao Y, Wu S*et al*. (2012) Identification of ten serum microRNAs from a genome‐wide serum microRNA expression profile as novel noninvasive biomarkers for nonsmall cell lung cancer diagnosis. Int J Cancer 130, 1620–1628. 21557218 10.1002/ijc.26177

[13] Wang C, Ding M, Xia M, Chen S, Van Le A, Soto‐Gil R, Shen Y, Wang N, Wang J, Gu W*et al*. (2015) A Five‐miRNA panel identified from a multicentric case‐control study serves as a novel diagnostic tool for ethnically diverse non‐small‐cell lung cancer patients. EBioMedicine 2, 1377–1385. 26629532 10.1016/j.ebiom.2015.07.034 PMC4634198

[14] Wang Y, Gu J, Roth JA, Hildebrandt MAT, Lippman SM, Ye Y, Minna JD & Wu X (2013) Pathway‐based serum microRNA profiling and survival in patients with advanced stage non‐small cell lung cancer. Cancer Res 73, 4801–4809. 23774211 10.1158/0008-5472.CAN-12-3273 PMC3760306

[15] Thind A & Wilson C (2016) Exosomal miRNAs as cancer biomarkers and therapeutic targets. J Extracell Vesicles 5, 31292. 27440105 10.3402/jev.v5.31292 PMC4954869

[16] Zhao C, Sun X & Li L (2019) Biogenesis and function of extracellular miRNAs. ExRNA 1, 38.

[17] Lane RE, Korbie D, Hill MM & Trau M (2018) Extracellular vesicles as circulating cancer biomarkers: opportunities and challenges. Clinical and translational medicine 7, 14. 29855735 10.1186/s40169-018-0192-7 PMC5981152

[18] Cazzoli R, Buttitta F, Di Nicola M, Malatesta S, Marchetti A, Rom WN & Pass HI (2013) microRNAs derived from circulating exosomes as noninvasive biomarkers for screening and diagnosing lung cancer. J Thorac Oncol 8, 1156–1162. 23945385 10.1097/JTO.0b013e318299ac32 PMC4123222

[19] Jin X, Chen Y, Chen H, Fei S, Chen D, Cai X, Liu L, Lin B, Su H, Zhao L*et al*. (2017) Evaluation of tumor‐derived exosomal miRNA as potential diagnostic biomarkers for early‐stage non‐small cell lung cancer using next‐generation sequencing. Clin Cancer Res 23, 5311–5319. 28606918 10.1158/1078-0432.CCR-17-0577

[20] Dejima H, Iinuma H, Kanaoka R, Matsutani N & Kawamura M (2017) Exosomal microRNA in plasma as a non‐invasive biomarker for the recurrence of non‐small cell lung cancer. Oncol Lett 13, 1256–1263. 28454243 10.3892/ol.2017.5569 PMC5403401

[21] Liu Q, Yu Z, Yuan S, Xie W, Li C, Hu Z, Xiang Y, Wu N, Wu L, Bai L*et al*. (2017) Circulating exosomal microRNAs as prognostic biomarkers for non‐small‐cell lung cancer. Oncotarget 8, 13048–13058. 28055956 10.18632/oncotarget.14369 PMC5355076

[22] Silva J, Garcia V, Zaballos A, Provencio M, Lombardia L, Almonacid L, Garcia JM, Dominguez G, Pena C, Diaz R*et al*. (2011) Vesicle‐related microRNAs in plasma of nonsmall cell lung cancer patients and correlation with survival. The European respiratory journal 37, 617–623. 20595154 10.1183/09031936.00029610

[23] Konoshenko MY, Lekchnov EA, Vlassov AV & Laktionov PP (2018) Isolation of extracellular vesicles: general methodologies and latest trends. Biomed Res Int 2018, 8545347. 29662902 10.1155/2018/8545347 PMC5831698

[24] Ding M, Wang C, Lu X, Zhang C, Zhou Z, Chen X, Zhang C‐Y, Zen K & Zhang C (2018) Comparison of commercial exosome isolation kits for circulating exosomal microRNA profiling. Anal Bioanal Chem 410, 3805–3814. 29671027 10.1007/s00216-018-1052-4

[25] Wan S, Wang J, Wang J, Wu J, Song J, Zhang C‐Y, Zhang C, Wang C & Wang J‐J (2017) Increased serum miR‐7 is a promising biomarker for type 2 diabetes mellitus and its microvascular complications. Diabetes Res Clin Pract 130, 171–179. 28646700 10.1016/j.diabres.2017.06.005

[26] Li D, , Bao P, Yin Z, Sun L, Feng J, He Z, Jin M & Liu C (2018) Exploration of the involvement of LncRNA in HIV‐associated encephalitis using bioinformatics. PeerJ 6, e5721. 30345171 10.7717/peerj.5721 PMC6187992

[27] Yu H, Guan Z, Cuk K, Brenner H & Zhang Y (2018) Circulating microRNA biomarkers for lung cancer detection in Western populations. Cancer Med 7, 4849–4862. 30259714 10.1002/cam4.1782 PMC6198213

[28] Halvorsen AR, Bjaanæs M, LeBlanc M, Holm AM, Bolstad N, Rubio L, Peñalver JC, Cervera J, Mojarrieta JC, López‐Guerrero JA*et al*. (2016) A unique set of 6 circulating microRNAs for early detection of non‐small cell lung cancer. Oncotarget 7, 37250–37259. 27191990 10.18632/oncotarget.9363 PMC5095073

[29] D'Souza‐Schorey C & Clancy JW (2012) Tumor‐derived microvesicles: shedding light on novel microenvironment modulators and prospective cancer biomarkers. Genes Dev 26, 1287–1299. 22713869 10.1101/gad.192351.112 PMC3387656

[30] Tang CP, Zhou H‐J, Qin J, Luo Y & Zhang T (2017) MicroRNA‐520c‐3p negatively regulates EMT by targeting IL‐8 to suppress the invasion and migration of breast cancer. Oncol Rep 38, 3144–3152. 29048659 10.3892/or.2017.5968

[31] Miao HL, Lei C‐J, Qiu Z‐D, Liu Z‐K, Li R, Bao S‐T & Li M‐Y (2014) MicroRNA‐520c‐3p inhibits hepatocellular carcinoma cell proliferation and invasion through induction of cell apoptosis by targeting glypican‐3. Hepatol Res 44, 338–348. 23607462 10.1111/hepr.12121

[32] Hu S, Chen H, Zhang Y, Wang C, Liu K, Wang H & Luo J (2017) MicroRNA‐520c inhibits glioma cell migration and invasion by the suppression of transforming growth factor‐beta receptor type 2. Oncol Rep 37, 1691–1697. 28184932 10.3892/or.2017.5421

[33] Li X, Fu Q, Li H, Zhu L, Chen W, Ruan T, Xu W & Yu X (2019) MicroRNA‐520c‐3p functions as a novel tumor suppressor in lung adenocarcinoma. Febs j 286, 2737–2752. 30942957 10.1111/febs.14835

[34] Xu X, Lu X, Sun J & Shu Y (2010) microRNA expression profiling of side population cells in human lung cancer and preliminary analysis. Zhongguo Fei Ai Za Zhi 13, 665–669. 20673480 10.3779/j.issn.1009-3419.2010.07.02 PMC6000378

[35] George JN, Thoi Ll, McManus LM & Reimann TA (1982) Isolation of human platelet membrane microparticles from plasma and serum. Blood 60, 834–840. 7115953

[36] Doyle LM & Wang MZ (2019) Overview of extracellular vesicles, their origin, composition, purpose, and methods for exosome isolation and analysis. Cells 8, 727. 10.3390/cells8070727 PMC6678302 31311206

