# Supplementary material for: Discovery and validation of extracellular vesicle‐associated miRNAs as noninvasive detection biomarkers for early‐stage non‐small‐cell lung cancer
Source: Mol Oncol. 2021 Jan 6;15(9):2439–52. doi: 10.1002/1878-0261.12889 (PMC8410569; doi:10.1002/1878-0261.12889)
Supplement: Supplementary file 1 — Table S1. Demographic and clinical features of the NSCLC patients and controls of the TLDA samples*. Table S2. Upregulated EV miRNAs in NSCLC pooled serum sample compared to normal control sample determined by TaqMan Low Density Assay. Table S3. Average expression level of the individual miRNA in different subtype of NSCLC and benign nodules*. Table S4. Average expression level of the individual miRNA in different NSCLC stage and the differences between stages*. Table S5. Areas under the curve and the asymptotic 95% confidence intervals of the individual miRNA, the panel of two‐miRNA in the different sets. Table S6. Areas under the curve and the asymptotic 95% confidence intervals of the individual miRNA, the panel of two‐miRNA in the different groups. Table S7. Univariate and multivariate logistic regression analyses of parameters associated with NSCLC in all NSCLC Cases*. Table S8. Demographic and clinical features of the NSCLC patients and controls for evaluation of miRNA levels in EVs and EV‐free fraction*. [file MOL2-15-2439-s001.doc]

**Supplemental materials**

**Multicentre Discovery and Validation of Extracellular Vesicles-associated miRNAs as Non-invasive Detection Biomarkers for Early-stage Non-Small-Cell Lung Cancer**

Yujie Zhong1，2, Xiaoyu Ding1，2, Yuying Bian1, Jing Wang1, Wanqing Zhou3, Xiangdong Wang4, Pumin Li2, Jun-Jun Wang1, Jing Li2*, Chunni Zhang1，2* and Cheng Wang1，2*

1Department of Clinical Laboratory, Jinling Hospital, State Key Laboratory of Analytical Chemistry for Life Science, Nanjing University School of Medicine, Nanjing University, Nanjing, 210002, China.

2 State Key Laboratory of Pharmaceutical Biotechnology, Jiangsu Engineering Research Center for MicroRNA Biology and Biotechnology, NJU Advanced Institute for Life Sciences (NAILS), Nanjing University School of Life Sciences, Nanjing University, Nanjing, 210046, China.

3Department of Laboratory Medicine, Nanjing Drum Tower Hospital, the Affiliated Hospital of Nanjing University Medical School, Nanjing, 210023, China.

4Department of Laboratory Medicine, the Affiliated Chest Hospital of Nanjing Medical School, Nanjing, 210029, China.

Yujie Zhong, Xiaoyu Ding and Yuying Bian contributed equally to this work.

* Corresponding authors.

Corresponding authors at: Department of Clinical Laboratory, Jinling Hospital, 305 East Zhongshan Rd., Nanjing, 210002, Jiangsu, China. Fax: 86-25-83686234; School of Life Sciences, Nanjing University, 163 Xianlin Rd., Nanjing 210046, China.

Running Title: Extracellular vesicles-associated miRNAs in non-small-cell lung cancer

E-mail addresses: wangcheng919@smail.nju.edu.cn (Cheng Wang), zchunni27@hotmail. com (Chunni Zhang), jingli220@nju.edu.cn (Jing Li).

**Supplementary Table 1.** Demographic and clinical features of the NSCLC patients and controls of the TLDA samples*.

| **Variable** | **Control** | | |  | **NSCLC** |  |
| --- | --- | --- | --- | --- | --- | --- |
|  | **Control_1** | **Control_2** | **Control_3** | **NSCLC_1** | **NSCLC_2** | **NSCLC_3** |
| **Number** | N = 12 | N = 12 | N = 12 | N = 12 | N = 12 | N = 12 |
| **Age** | 63.8 ± 12.1 | 56.1 ± 16.0 | 57.3 ± 16.8 | 60.6 ± 14.2 | 58.4 ± 13.1 | 60.8 ± 11.1 |
| **≤ 59** | 4 | 7 | 5 | 5 | 6 | 5 |
| **> 59** | 8 | 5 | 7 | 7 | 6 | 7 |
| **Sex** |  |  |  |  |  |  |
| **Male** | 12 | 12 | 0 | 8 | 8 | 6 |
| **Female** | 0 | 0 | 12 | 4 | 4 | 6 |
| **Smoking status** | |  |  |  |  |  |
| **Ever and current** | 4 | 5 | 3 | 6 | 5 | 4 |
| **Never** | 8 | 7 | 9 | 6 | 7 | 8 |
| **Tumor subtype** | |  |  |  |  |  |
| **AC** | - | - | - | 12 | 9 | 10 |
| **SCC** | - | - | - | 0 | 3 | 2 |
| **Tumor stage** | |  |  |  |  |  |
| **I** | - | - | - | 1 | 2 | 1 |
| **II** | - | - | - | 1 | 2 | 3 |
| **III** | - | - | - | 6 | 5 | 4 |
| **IV** | - | - | - | 4 | 3 | 4 |
| * The data are expressed as the mean (SD). | | | | |  |  |

**Supplementary Table 2.** Upregulated EV miRNAs in NSCLC pooled serum sample compared to normal control sample determined by TaqMan Low Density Assay.

| **miRNA** | **Control** | | **NSCLC** | | **Fold change** |
| --- | --- | --- | --- | --- | --- |
|  | **Mean** | **SD** | **Mean** | **SD** |  |
| hsa-miR-190b | 2.669756914 | 3.652153627 | 166065.8048 | 287625.5867 | 62202.59 |
| hsa-miR-1305 | 0.201491479 | 0.34870728 | 4976.142629 | 8618.430435 | 24696.54 |
| hsa-miR-206 | 0.002127839 | 0.000887903 | 15.38091909 | 20.41941711 | 7228.42 |
| hsa-miR-573 | 0.000238148 | 0.000152548 | 1.483133624 | 1.317082585 | 6227.79 |
| dme-miR-7 | 0.000238148 | 0.000152548 | 0.363753759 | 0.616582755 | 1527.43 |
| hsa-miR-409-3p | 0.005908502 | 0.009418743 | 7.34937888 | 6.37079211 | 1243.87 |
| hsa-miR-30e-3p | 0.012607582 | 0.017329543 | 9.00695145 | 15.5966421 | 714.41 |
| hsa-miR-181a | 0.000195543 | 3.55392E-05 | 0.089749577 | 0.081653318 | 458.98 |
| hsa-miR-571 | 0.007369832 | 0.012479008 | 2.434562686 | 2.06685793 | 330.34 |
| hsa-miR-31 | 0.000195543 | 3.55392E-05 | 0.040718973 | 0.050094054 | 208.24 |
| hsa-miR-193b | 0.000195543 | 3.55392E-05 | 0.034043098 | 0.029497159 | 174.10 |
| hsa-miR-1298 | 0.000238148 | 0.000152548 | 0.032152733 | 0.027747859 | 135.01 |
| hsa-miR-212 | 0.000195543 | 3.55392E-05 | 0.012801315 | 0.011882102 | 65.47 |
| hsa-miR-1274b | 0.332089185 | 0.25026461 | 18.76389315 | 28.98616148 | 56.50 |
| hsa-miR-203 | 0.001546429 | 0.00230635 | 0.055155702 | 0.041751967 | 35.67 |
| hsa-miR-127-3p | 0.002631713 | 0.00424669 | 0.069500758 | 0.060708193 | 26.41 |
| hsa-miR-422a | 0.000195543 | 3.55392E-05 | 0.004307946 | 0.003408181 | 22.03 |
| hsa-miR-720 | 0.086171572 | 0.118083423 | 1.351720388 | 0.708534911 | 15.69 |
| hsa-miR-142-5p | 0.000195543 | 3.55392E-05 | 0.00265078 | 0.003571134 | 13.56 |
| hsa-miR-590-5p | 0.000195543 | 3.55392E-05 | 0.002437302 | 0.003094002 | 12.46 |
| hsa-miR-136 | 0.007203627 | 0.01210488 | 0.063496983 | 0.10883838 | 8.81 |
| hsa-miR-645 | 0.023137383 | 0.022219433 | 0.199389204 | 0.312821777 | 8.62 |
| hsa-miR-601 | 0.011367338 | 0.012109884 | 0.08116949 | 0.103413376 | 7.14 |
| hsa-miR-374a | 0.002111974 | 0.000871581 | 0.012403727 | 0.02053434 | 5.87 |
| hsa-miR-15b | 0.004475789 | 0.002787179 | 0.026020344 | 0.026016745 | 5.81 |
| hsa-miR-518f | 0.003931839 | 0.000973645 | 0.022328079 | 0.028585535 | 5.68 |
| hsa-miR-520c-3p | 0.600696994 | 1.040151508 | 2.323389897 | 2.195478838 | 3.87 |
| hsa-miR-34a | 237.1761647 | 241.6516132 | 864.6760371 | 764.1407702 | 3.65 |
| hsa-miR-30a-5p | 0.469692123 | 0.363927178 | 1.669694152 | 1.983077213 | 3.55 |
| hsa-miR-302c | 0.194523959 | 0.227330042 | 0.675076366 | 1.044683635 | 3.47 |
| hsa-miR-208 | 185.9835044 | 212.6021456 | 612.6435597 | 591.6785285 | 3.29 |
| hsa-miR-126^#^ | 0.002723754 | 0.003407667 | 0.008105104 | 0.002232753 | 2.98 |
| hsa-miR-548c-3p | 0.002045869 | 0.001616811 | 0.005880677 | 0.006883394 | 2.87 |
| hsa-miR-26b | 0.012840424 | 0.011549437 | 0.03446702 | 0.053364553 | 2.68 |
| hsa-miR-520d-5p | 0.001337811 | 0.002005655 | 0.002753254 | 0.002895353 | 2.06 |
| hsa-miR-17 | 0.514584229 | 0.447265851 | 1.047422946 | 1.627542088 | 2.04 |
| hsa-miR-151-3p | 0.002255565 | 0.000237911 | 0.004577862 | 0.003815269 | 2.03 |

**Supplementary Table 3.** Average expression level of the individual miRNA in different subtype of NSCLC and benign nodules*.

| **miRNA** | **Stage** | **Number** | **Average relative**  **concentration** | **Fold-change (comparison with controls)** | **Fold-change (comparison with benign nodules)** | **P value** |
| --- | --- | --- | --- | --- | --- | --- |
| **miR-520c-3p** | Control | 120 | 0.282(0.002) |  |  |  |
|  | Benign nodules | 31 | 0.276(0.011) | 0.979 |  | 0.0528 |
|  | AC | 132 | 0.697(0.004) | 2.472 | 2.681 | <0.001 |
|  | SCC | 27 | 0.818(0.024) | 2.901 | 2.964 | <0.001 |
| **miR-1274b** | Control | 120 | 2.355(0.015) |  |  |  |
|  | Benign nodules | 31 | 3.059(0.079) | 1.230 |  | 0.1423 |
|  | AC | 132 | 5.445(0.030) | 2.312 | 1.780 | <0.001 |
|  | SCC | 27 | 4.958(0.183) | 2.105 | 1.621 | <0.001 |

* The relative concentrations of the miRNAs were normalized to cel-miR-39 and presented as the mean (SE), and the differences between the different group were analyzed by Kruskal-Wallis test.

**Supplementary Table 4.** Average expression level of the individual miRNA in different NSCLC stage and the differences between stages*.

| **miRNA** | **Stage** | **Number** | | **Average relative**  **concentration** | **Fold-change (comparison with controls)** | **Fold-change (comparison with benign nodules)** | **P value** |
| --- | --- | --- | --- | --- | --- | --- | --- |
| **miR-520c-3p** | Control | | 120 | 0.282(0.002) |  |  |  |
|  | Benign nodules | | 31 | 0.276(0.011) | 0.979 |  |  |
|  | NSCLC stage I | | 96 | 0.706 (0.006) | 2.504 | 2.55 | <0.001 |
|  | NSCLC stage II | | 23 | 0.751 (0.024) | 2.663 | 2.72 | <0.001 |
|  | NSCLC stage III | | 32 | 0.746 (0.016) | 2.645 | 2.70 | <0.001 |
|  | NSCLC stage IV | | 6 | 0.736 (0.089) | 2.610 | 2.66 | <0.001 |
| **miR-1274b** | Control | | 120 | 2.355(0.015) |  |  |  |
|  | Benign nodules | | 31 | 3.059(0.079) | 1.230 |  |  |
|  | NSCLC stage I | | 96 | 5.084 (0.040) | 2.159 | 1.66 | <0.001 |
|  | NSCLC stage II | | 23 | 6.390 (0.183) | 2.713 | 2.09 | <0.001 |
|  | NSCLC stage III | | 32 | 6.960 (0.149) | 2.955 | 2.27 | <0.001 |
|  | NSCLC stage IV | | 6 | 5.697 (1.045) | 2.420 | 1.86 | <0.001 |

* The relative concentrations of the miRNAs were normalized to cel-miR-39 and presented as the mean (SE), and the differences between the different stages were analyzed by Kruskal-Wallis test.

**Supplementary Table 5.** Areas under the curve and the asymptotic 95% confidence intervals of the individual miRNA, the panel of two-miRNA in the different sets.

| **Set** | **miRNA** | **Area** | **Std. Error** | **Asymptotic Sig.** | **Asymptotic 95% Confidence Interval** | |
| --- | --- | --- | --- | --- | --- | --- |
|  |  |  |  |  | **Lower Bound** | **Upper Bound** |
| **Training set** | miR-520c-3p | 0.882 | 0.037 | < 0.001 | 0.809 | 0.955 |
|  | miR-1274b | 0.840 | 0.042 | < 0.001 | 0.757 | 0.924 |
|  | miR-Panel | 0.920 | 0.028 | < 0.001 | 0.865 | 0.976 |
| **Validation set** | miR-520c-3p | 0.790 | 0.058 | < 0.001 | 0.675 | 0.904 |
|  | miR-1274b | 0.814 | 0.055 | < 0.001 | 0.706 | 0.921 |
|  | miR-Panel | 0.861 | 0.047 | < 0.001 | 0.768 | 0.953 |
| **Testing set** | miR-520c-3p | 0.842 | 0.049 | < 0.001 | 0.746 | 0.939 |
|  | miR-1274b | 0.895 | 0.044 | < 0.001 | 0.808 | 0.982 |
|  | miR-Panel | 0.929 | 0.030 | < 0.001 | 0.870 | 0.988 |
|  |  |  |  |  |  |  |

**Supplementary Table 6.** Areas under the curve and the asymptotic 95% confidence intervals of the individual miRNA, the panel of two-miRNA in the different groups.

| **Set** | **miRNA** | **Area** | **Std. Error** | **Asymptotic Sig.** | **Asymptotic 95% Confidence Interval** | | |
| --- | --- | --- | --- | --- | --- | --- | --- |
|  |  |  |  |  | **Lower Bound** | **Upper Bound** |  |
| **All NSCLC patients** | miR-520c-3p | 0.819 | 0.025 | < 0.001 | 0.770 | 0.867 |  |
|  | miR-1274b | 0.788 | 0.027 | < 0.001 | 0.736 | 0.841 |  |
|  | miR-Panel | 0.857 | 0.022 | < 0.001 | 0.813 | 0.901 |  |
| **NSCLC stage I** | miR-520c-3p | 0.818 | 0.029 | < 0.001 | 0.761 | 0.874 |  |
|  | miR-1274b | 0.758 | 0.032 | < 0.001 | 0.695 | 0.822 |  |
|  | miR-Panel | 0.845 | 0.026 | < 0.001 | 0.793 | 0.896 |  |
| **NSCLC stage II** | miR-520c-3p | 0.837 | 0.041 | < 0.001 | 0.757 | 0.918 |  |
|  | miR-1274b | 0.832 | 0.042 | < 0.001 | 0.749 | 0.914 |  |
|  | miR-Panel | 0.904 | 0.027 | < 0.001 | 0.852 | 0.957 |  |
| **NSCLC stage III** | miR-520c-3p | 0.820 | 0.043 | < 0.001 | 0.736 | 0.904 |  |
|  | miR-1274b | 0.861 | 0.035 | < 0.001 | 0.792 | 0.929 |  |
|  | miR-Panel | 0.879 | 0.033 | < 0.001 | 0.814 | 0.944 |  |
| **NSCLC stage IV** | miR-520c-3p | 0.754 | 0.124 | 0.036 | 0.510 | 0.998 |  |
|  | miR-1274b | 0.715 | 0.105 | 0.076 | 0.510 | 0.920 |  |
|  | miR-Panel | 0.754 | 0.124 | 0.036 | 0.510 | 0.998 |  |
| **NSCLC stage II-IV** | miR-520c-3p | 0.820 | 0.033 | < 0.001 | 0.756 | 0.884 |  |
|  | miR-1274b | 0.835 | 0.030 | < 0.001 | 0.776 | 0.895 |  |
|  | miR-Panel | 0.881 | 0.026 | < 0.001 | 0.831 | 0.932 |  |
| **NSCLC-AC** | miR-520c-3p | 0.828 | 0.026 | < 0.001 | 0.778 | 0.878 |  |
|  | miR-1274b | 0.778 | 0.029 | < 0.001 | 0.722 | 0.834 |  |
|  | miR-Panel | 0.858 | 0.023 | < 0.001 | 0.812 | 0.903 |  |
| **NSCLC-SCC** | miR-520c-3p | 0.773 | 0.056 | < 0.001 | 0.662 | 0.884 |  |
|  | miR-1274b | 0.841 | 0.041 | < 0.001 | 0.761 | 0.921 |  |
|  | miR-Panel | 0.861 | 0.039 | < 0.001 | 0.785 | 0.938 |  |
| **Malignant lesions *VS* benign nodules** | miR-520c-3p | 0.823 | 0.047 | < 0.001 | 0.730 | 0.915 |  |
|  | miR-1274b | 0.713 | 0.052 | < 0.001 | 0.611 | 0.815 |  |
|  | miR-Panel | 0.823 | 0.047 | < 0.001 | 0.730 | 0.915 |  |

**Supplementary Table 7.** Univariate and multivariate logistic regression analyses of parameters associated with NSCLC in all NSCLC Cases*.

| **Factor** | **Univariate analysis** | | | | **Multivariate analysis** | | | |
| --- | --- | --- | --- | --- | --- | --- | --- | --- |
|  | **OR** | **95% CI** | | ***P*-value** | **OR** | **95% CI** | | ***P-*value** |
| **Age (< 60 / ≥ 60)** | 1.093 | 0.680 | 1.093 | 0.713 | 1.228 | 0.684 | 2.205 | 0.492 |
| **Sex (male / female)** | 1.171 | 0.727 | 1.880 | 0.516 | 0.743 | 0.346 | 1.596 | 0.446 |
| **Smoking status (Ever and Current / Never)** | 1.703 | 1.020 | 2.842 | 0.042 | 2.152 | 0.96 | 4.824 | 0.063 |
| **miR-520c-3p (low / high)** | 10.048 | 4.153 | 24.312 | < 0.001 | 3.558 | 1.287 | 9.839 | 0.014 |
| **miR-1274b (low / high)** | 9.771 | 4.036 | 23.655 | < 0.001 | 6.544 | 2.486 | 17.226 | < 0.001 |
| **Risk score (low ≤ 1.957 / high > 1.957)** | 16.128 | 6.702 | 38.813 | < 0.001 | 12.238 | 4.86 | 30.82 | < 0.001 |

* OR and 95% CI were missed in univariate logistic regression analyses because the risk score at least one group are all positive/negative and value can’t be generated.

**Supplementary Table 8.** Demographic and clinical features of the NSCLC patients and controls for evaluation of miRNA levels in EVs and EV-free fraction*.

| **Variable** | **Control** | | |  | **NSCLC** | **P-value** |
| --- | --- | --- | --- | --- | --- | --- |
|  | **N =12** | | | **N =12** | | |
| **Age** | 61.2 ± 4.2 | | | 64.8 ± 15.6 0.116^a^ | | |
| **≤ 59** | 5 | | | 3 0.387^b^ | | |
| **> 59** | 7 | | | 9 | | |
| **Sex** |  |  |  |  |  | 0.615^b^ |
| **Male** | 9 |  |  | 10 |  |  |
| **Female** | 3 |  |  | 2 |  |  |
| **Tumor subtype** | |  |  |  |  |  |
| **AC** | - | - | - | 11 |  |  |
| **SCC** | - | - | - | 1 |  |  |
| **Tumor stage** | |  |  |  |  |  |
| **I** | - | - | - | 5 |  |  |
| **II** | - | - | - | 1 |  |  |
| **III** | - | - | - | 5 |  |  |
| **IV** | - | - | - | 1 |  |  |
| ^*^ The data are expressed as the mean (SD).  ^a^ P-values were measured by Mann–Whitney independent t test.  ^b^ P-values were measured by two sides χ^2^ test. | | | | |  |  |
